# Health Benefits of Traditional Sage and Peppermint Juices: Simple Solutions for Antioxidant and Antidiabetic Support

**DOI:** 10.3390/foods14071182

**Published:** 2025-03-28

**Authors:** Sanja Krstić, Isidora Milanović, Nebojša Stilinović, Saša Vukmirović, Nebojša Pavlović, Sanja Berežni, Milena Rašeta

**Affiliations:** 1 Institute of Pharmaceutical Sciences, University of Graz, Beethovenstraße 8, 8010 Graz, Austria; 2 Department of Pharmacology, Biochemistry, Pharmacy and Ecology, College of Health Sciences, Academy for Applied Studies Belgrade, Cara Dušana 254, 11080 Belgrade, Serbia; isidora.milanovic@assb.edu.rs; 3 Department of Pharmacology, Toxicology and Clinical Pharmacology, Faculty of Medicine, University of Novi Sad, Hajduk Veljkova 3, 21000 Novi Sad, Serbia; nebojsa.stilinovic@gmail.com (N.S.); sasavukmirovic99@gmail.com (S.V.); 4 Department of Pharmacy, Faculty of Medicine, University of Novi Sad, Hajduk Veljkova 3, 21000 Novi Sad, Serbia; nebojsa.pavlovic@mf.uns.ac.rs; 5 Department of Chemistry, Biochemistry, and Environmental Protection, Faculty of Sciences, University of Novi Sad, Trg Dositeja Obradovića 3, 21000 Novi Sad, Serbia; sanja.beric@dh.uns.ac.rs (S.B.); milena.raseta@dh.uns.ac.rs (M.R.)

**Keywords:** bioactive compounds, functional drinks, glucose regulation, herbal beverages, *Mentha piperita*, metabolic health, polyphenols, *Salvia officinalis*

## Abstract

Sage and peppermint leaves and flowers possess antioxidant, anti-inflammatory, and metabolic-regulating properties. This study compared the phenolic profiles confirmed using LC-MS/MS analysis and the biological activities of traditionally prepared sage (SJ) and peppermint juices (PJ), evaluating their *ex vivo* antioxidant enzyme activity and antidiabetic potential in experimental mice. Quinic acid (2571.86 ± 1.15 µg/g dry weight (d.w.)) and apigenin-7-*O*-β-glucoside (324.36 ± 1.15 µg/g d.w.) were the predominant phenolic compounds in SJ, while PJ contained caffeic acid (16.96 ± 0.12 µg/g d.w.) and quinic acid (184.27 ± 0.45 µg/g d.w.). The administration of SJ and PJ for ten days significantly reduced the blood glucose levels of the mice. Compared to the control group, which showed an increase from 15.89 ± 3.45 to 29.54 ± 8.94 mmoL/L, SJ-20 mg/kg body weight (BW)-treated mice exhibited a more moderate rise (16.25 ± 7.33 to 21.50 ± 10.38 mmoL/L). Juice administration also enhanced antioxidant enzyme activity, with PJ-20 mg/kg BW significantly increasing superoxide dismutase (SOD) activity (30.15 ± 3.99 U/mg proteins) compared to the control (18.83 ± 2.04 U/mg proteins). Additionally, catalase (CAT) activity was elevated, indicating enhanced oxidative stress defense mechanisms. Furthermore, treatment with SJ-20 mg/kg BW reduced aspartate aminotransferase (AST) (7025.8 ± 1038.8 to 2782 ± 426.9 IU/L) and alanine aminotransferase (ALT) levels (10679 ± 1409.2 to 5336.5 ± 801.5 IU/L), suggesting hepatoprotective effects. These results support the traditional use of sage and peppermint juices as functional beverages with antioxidant, antidiabetic, and hepatoprotective properties, warranting further clinical investigation.

## 1. Introduction

Sage and peppermint belong to the Lamiaceae family and have been cultivated worldwide for their culinary and medicinal properties [1,2,3]. These herbs are widely utilized in traditional medicine and food preparation due to their rich composition of biologically active compounds [2,3,4,5]. They are commonly consumed as teas, tinctures, or culinary additives, with documented health-promoting effects. Experimental studies have confirmed various pharmacological activities of sage and peppermint extracts, including antioxidant, anti-inflammatory, antimicrobial, and metabolic regulatory effects [6,7]. Notably, the antioxidant capacity of their extracts has been reported to be comparable to, or even greater than, that of certain synthetic antioxidants [8].

Sage (*Salvia officinalis* L.), a perennial subshrub native to the Mediterranean region [9], is highly valued for its essential oils. The various parts of *S. officinalis*, rich in bioactive compounds such as flavonoids, alkaloids, tannins, and glycosides, play a crucial role in inhibiting pathogenic bacteria and alleviating different pathologies [7]. In addition, phenolic acids and flavonoids are the major bioactive compounds in sage, contributing to its diverse pharmacological properties [1,4,9,10,11,12,13]. These include anti-inflammatory, antioxidant, antiparasitic, enzyme inhibitory, anti-tumor, antidiabetic, neuroprotective, gastroprotective, immunomodulatory, and antimicrobial activities [1,4,6,12,13,14,15,16,17,18]. Key bioactive compounds such as carnosol, carnosic acid, and rosmarinic acid have been identified as the primary contributors to these effects [19].

Peppermint (*Mentha piperita* L.), recognized for its pleasant aroma and medicinal properties, is widely used in the food, pharmaceutical, and cosmetic industries [7,20]. Its aerial parts (leaves) are rich in various classes of phenolics, including flavones (e.g., luteolin derivatives), flavonols (e.g., catechin, quercetin), flavanones (e.g., eriocitrin derivatives), phenolic acids (e.g., rosmarinic and caffeic acids), lignans, and stilbenes, as well as some antioxidant vitamins, including carotenoids and ascorbic acid, which exhibit significant antioxidant, antidiabetic, anticancer, anti-inflammatory, anti-hyperlipidemic, and cardioprotective activities [3,20,21,22,23,24,25,26]. Additionally, a recent study by Al-Mijalli et al. [26] used gas chromatography–mass spectrometry (GC-MS) analysis to identify volatile compounds in Moroccan *M. piperita* leaves and found that carvone (70.25%) was the most dominant compound, followed by neodihydrocarveol (4.22%) and β-myrcene (4.00%). Traditionally, peppermint extracts have been used for digestive disorders, nausea, bronchitis, and anorexia [27]. Moreover, their antiviral activity has been demonstrated against various viruses, including influenza A, herpes simplex virus, and HIV-1 [23].

Environmental factors significantly influence the phytochemical composition of sage and peppermint. Plants grown in coastal regions, such as Adriatic or Dalmatian sage, accumulate higher levels of essential oils and bioactive compounds due to greater sun exposure [4,28]. These climatic conditions enhance the therapeutic potential of the herbs, making them highly valued in traditional medicine [29].

The growing global demand for sustainably produced food has driven significant interest in natural food-derived antioxidants, including herbal extracts from sage and peppermint [30,31]. Although sage tea and extracts are known for their bitter and pungent taste, which may limit their widespread dietary use, peppermint juice is more commonly consumed due to its pleasant flavor [32]. However, traditional juice preparation methods often lead to low extraction efficiency of bioactive phenolic compounds, which may impact their potential health benefits.

Herbal drinks have long been an integral part of traditional medicine, particularly among elderly populations, where their use has been passed down through generations [33]. These beverages are deeply integrated into culinary traditions across several countries, including China, India, Indonesia, Malaysia, and Sri Lanka, where traditional medicines are widely used [33]. Historically, sage and peppermint juices have not been consumed daily, but intermittently, primarily for their health benefits, such as relieving coughs, alleviating pain, and promoting detoxification [16]. Rosmarinic acid, a dominant phenolic compound in sage and peppermint, is known for its strong antioxidant properties and has been associated with metabolic benefits, including potential antidiabetic effects [34]. Generally, herbal drinks, whether derived from a single plant or a combination of medicinal herbs, are rich in phytochemicals, including flavonoids, phenolic compounds, carotenoids, plant sterols, glucosinolates, alkaloids, polyacetylenes, saponins, and terpenoids, among others [33]. Conversely, poor dietary habits leading to malnutrition pose a significant challenge, contributing to metabolic disorders such as diabetes mellitus [3]. Diabetes is characterized by chronic hyperglycemia due to insufficient insulin production or impaired insulin function. It is now recognized as a global public health crisis, with conventional drug treatments often limited by side effects, accessibility, and cost [35]. In addition to their antidiabetic potential, these medicinal plants have been extensively studied for their antioxidant and hepatoprotective properties [1,6,7,13]. Their bioactive compounds can mitigate oxidative stress by enhancing endogenous antioxidant defense systems, including key enzymes such as superoxide dismutase (SOD), catalase (CAT), and glutathione peroxidase (GPx). Furthermore, tested medicinal plant extracts have demonstrated hepatoprotective effects by reducing liver damage markers and modulating inflammatory pathways [36].

In this context, the extensive use and economic importance of *S. officinalis* and *M. piperita*, attributed to their nutritional value and high content of bioactive phenolic compounds, suggest their potential as sugar substitutes in the future. Therefore, the incorporation of phytoconstituents from these two Lamiaceae species into functional foods may offer a cost-effective and biocompatible approach to diabetes management.

Despite extensive research on sage and peppermint teas and extracts, there is a notable gap in studies investigating the biological effects of their juices, particularly concerning metabolic disorders. The present study aims to characterize the composition of traditionally prepared sage and peppermint juices from plants collected in Montenegro (Balkan region) and to evaluate their antioxidant and antidiabetic properties *ex vivo*. To the best of our knowledge, this is the first *ex vivo* study that examines the antidiabetic potential of sage and peppermint juices in diabetic mice. These findings contribute to the growing body of evidence supporting the use of herb-derived beverages as functional foods with potential therapeutic applications for metabolic health management.

## 2. Materials and Methods

### 2.1. Plant Material Collection and Traditional Juice Preparation

Sage flowers and dried peppermint leaves, harvested in a village in Montenegro (42°20′14″ N 18°55′02″ E/42.337333° N 18.917166° E), were purchased from a local market in Cetinje, Montenegro. The fresh materials used for preparation were carefully weighed, with 100 g of dried plant material derived from approximately 800–1000 g of fresh plant material.

Traditional Juice Preparation:

A total of 100 g of dried plant material (sage flowers or peppermint leaves) was placed in a 2 L borosilicate glass bowl (DURAN^®^, DWK Life Sciences, Mainz, Germany) with 1 L of tap water and left to stand for 24 h, with occasional stirring. The mixtures were then filtered through a domestic strainer. Next, 1 kg of sugar and juice from two squeezed lemons (1.5 mL total) were added to 700 mL of the filtered mixture. The solution was stirred until the sugar dissolved and was then left to undergo continuous mixing for 24 h at room temperature (25 °C) using IKA Plate magnetic stirrers (RCT digital, IKA-Werke GmbH & Co. KG, Staufen im Breisgau, Germany), followed by an additional 5 h of standing. The mixtures were then sequentially filtered through gauze and Whatman Grade 595 qualitative filter paper (Cytiva, Marlborough, MA, USA) before being transferred into sterile DURAN^®^ GLS 80 wide-neck bottles (DWK Life Sciences, Mainz, Germany). The bottle lids were wrapped with parafilm, and the juices were stored in a dark place at 4 °C (humidity: 85%) until the experiment.

### 2.2. Liquid Chromatography–Mass Spectrometry (LC-MS/MS) Analysis

The chemical composition of traditional peppermint (PJ) and sage juice (SJ), focusing on bioactive compounds, was analyzed using LC-MS/MS. A total of 44 phenolic compounds, as well as quinic acid, were quantified based on the method outlined by Orčić et al. [37]. The analysis was conducted using an Agilent 1200 Series HPLC coupled with a 6410A Triple Quad MS (Agilent Technologies, Santa Clara, CA, USA), controlled by MassHunter software (version B.07.00). Standards from Sigma-Aldrich (Darmstadt, Hesse, Germany), Fluka Chemie (Darmstadt, Hesse, Germany), and ChromaDex (Los Angeles, CA, USA) were prepared in 50% methanol (25 mg/mL), and 5 μL injections were separated on a Zorbax Eclipse XDB-C18 column (Agilent Technologies, Santa Clara, CA, USA). Data were acquired in dynamic MRM mode and quantified using MassHunter. Calibration curves were generated with OriginPro software (version 2019b). Retention times and MS parameters are provided in Table S1.

### 2.3. Animal Treatment—Experimental Protocol

Study approval was granted by the Ethics Committee of the University of Novi Sad (Approval No. EK III-2018-01). The study adhered to the ARRIVE guidelines and was conducted in compliance with the U.K. Animals (Scientific Procedures) Act 1986, EU Directive 2010/63/EU for animal experiments. Male mice (*Mus musculus*, NMRI Haan strain), with body weights (BW) ranging from 30–40 g, were orally administered with SJ and PJ at doses of 20 mg/kg BW, 40 mg/kg BW, and 80 mg/kg BW once daily in the late afternoon for 10 days. During the study, all animals had free access to food and water and were kept on a 12 h light/dark cycle. Using a random selection method, the animals were distributed into 14 groups of 6 mice, including 2 control and 12 experimental groups, making a total of 84 animals (Table S2). The sample size was determined based on power analysis using G*Power 3.1.9.7. software to ensure statistical power of a minimum of 80%.

### 2.4. Antidiabetic Activity

The antidiabetic effects of SJ and PJ were evaluated using oral glucose tolerance test (OGTT) and streptozotocin-induced diabetes models.

#### 2.4.1. Oral Glucose Tolerance Test (OGTT)

Blood glucose levels were measured prior to glucose administration. A glucose solution (500 mg/kg BW) was administered orally, and blood glucose was measured 30 min after administration.

#### 2.4.2. Streptozotocin-Induced Diabetes

Blood glucose was measured before streptozotocin administration. Streptozotocin (150 mg/kg BW) was administered intraperitoneally (IP), and glycaemia measuring blood glucose was measured 72 h after administration. Diabetes was considered successfully induced if blood glucose levels exceeded 15 mmol/L.

Glycaemia (blood glucose level) in the mice was measured using capillary blood samples taken by a small incision near the tip of the tail. The test strip (ACCU CHEK Active, Roche, Basel, Switzerland) was drawn closer to the blood drop until it absorbed enough to begin the test.

### 2.5. Hepatoprotective Enzyme Activity

#### Aspartate and Alanine Aminotransferase Assays

Serum, harvested from blood samples, was used to analyze the enzymatic activity of aspartate (AST) and alanine (ALT) aminotransferase, which serve as markers of liver damage. The activities of AST and ALT were determined using the fully automated Olympus AU400 analyzer (Hamburg, Germany).

### 2.6. Ex Vivo Antioxidant Enzyme Activity and Lipid Peroxidation in the Liver

#### Paracetamol-Induced Oxidative Stress

In experimental groups C_1_ and SJ20a-PJ80a, after the final OGTT, the animals were treated with 130 mg/kg paracetamol to assess the antioxidant properties of SJ and PJ. Then, 24 h after paracetamol administration, the animals were euthanized, and blood and liver tissue samples were collected.

Oxidative status in the liver was evaluated by measuring lipid peroxidation (LPx) and the activities of oxidative stress-related enzymes, including superoxide-dismutase (SOD), catalase (CAT), glutathione peroxidase (GPx), glutathione reductase (GR), and glutathione-S-transferase (GST) in liver homogenates, using the spectrophotometric methods described by Rašeta et al. [38].

All measurements were taken using a Multiskan Ex spectrophotometer, Thermo Fisher Scientific, Waltham, MA, USA.

### 2.7. Statistical Analysis

Statistical analysis was conducted using IBM SPSS statistical software (version 23.0) (IBM Corporation, Armonk, NY, USA). Data are presented as the mean ± standard deviation (SD). For the comparison of blood glucose levels and antioxidant enzyme activity between experimental groups, either analysis of variance (ANOVA) or the Kruskal–Wallis test was used. Post hoc analysis for ANOVA was performed with Tukey’s test, while the Mann–Whitney U test was applied following the Kruskal–Wallis test. A *p*-value of less than 0.05 (*p* < 0.05) was considered statistically significant.

## 3. Results and Discussion

### 3.1. LC-MS/MS Profiling of Traditionally Prepared Sage and Peppermint Juices

Based on the results presented in Table 1, among the 44 tested phenolic compounds and quinic acid, a total of seven compounds were quantified in SJ. In contrast, PJ contained only two quantified compounds—one hydroxycinnamic acid and quinic acid—while the concentrations of other detected compounds were above the limit of detection (LOD) but below the limit of quantification (LOQ). The most abundant compounds in both juices were quinic acid and caffeic acid, with significantly higher concentrations in SJ samples (2571.86 ± 1.15 µg/g dry weight (d.w.) and 154.98 ± 0.01 µg/g d.w., respectively).

Numerous studies have reported the phenolic profiles of sage and peppermint in various forms, including tea, extracts, essential oils, infusions, and tinctures, highlighting their health benefits [7,10,11,15,24]. While phenolic content varies across samples, to the best of our knowledge, no data are available on the phenolic composition of SJ and PJ.

The results obtained in this study indicate that a cyclohexanecarboxylic acid, quinic acid (SJ: 2571.86 ± 1.15 µg/g d.w. and PJ: 184.27 ± 0.45 µg/g d.w.), was the most abundant bioactive compound in both tested samples, followed by caffeic acid (SJ: 154.98 ± 0.01 µg/g d.w. and PJ: 16.96 ± 0.12 µg/g d.w.). Among the tested flavonoids, flavone apigenin 7-*O*-glucoside was the second most abundant compound in SJ, with a concentration of 324.36 ± 1.15 µg/g d.w., though it was not quantified in PJ, as its amount was above the LOD but below the LOQ. This flavonoid glucoside has been detected in *S. officinalis* samples in previous studies but not in juices, as stated above [15,39]. In contrast, its aglycone, apigenin, was quantified in significantly lower amounts (16.40 ± 1.01 µg/g d.w.) in the same sample, SJ. Similarly, luteolin-7-*O*-glucoside was found at 110.72 ± 0.02 µg/g d.w. in SJ, a concentration that is lower than apigenin-7-*O*-glucoside but still notable. Additionally, it was not detectable in PJ, as indicated by <LOQ in Table 1.

These results confirm that both analyzed juices, SJ and PJ, are significant sources of bioactive phenolic compounds. The data presented in Table 1 align with those of previous studies that primarily investigated *S. officinalis* and *M. piperita* leaves in various forms, including teas, extracts, essential oils, infusions, and commercially available tinctures. Among these, caffeic acid was the most prevalent compound in *S. officinalis* [6,10,11,15] and *M. pipperita* [24,40,41]. This hydroxycinnamic acid is primarily quantified in leaves, except for in a study by Esmaili et al. [40], which analyzed a 70% ethanolic extract of stems and hairy roots. Compared to the available literature, quinic acid has only recently been confirmed in *S. officinalis* [13], where it was detected using RP-HPLC analysis in methanolic leaf extracts.

Notably, our study is the first to report the presence of chrysoeriol and quercetin-3-*O*-glucoside in both SJ and PJ. In SJ, their respective concentrations were 40.68 ± 0.25 µg/g d.w. and 3.29 ± 0.13 µg/g d.w., whereas in PJ, their levels were below the limit of quantification (LOQ) but above the limit of detection (LOD) (Table 1).

Rosmarinic acid has been highlighted as the most common phenolic compound in both plant species [3,7,9,25,40,41], followed by luteolin-7-*O*-glucoside [15,24,41], apigenin-7-*O*-glucoside [9,11,15,24], and apigenin [9,13,15,24]. These biomolecules are well known for their potent antioxidant activity, primarily due to their ability to neutralize free radical species [42]. Additionally, they have demonstrated anticancer, antidiabetic, and antibacterial properties [4,14].

The content of quinic acid in SJ (2571.86 ± 1.15 µg/g d.w.) was significantly higher than in PJ (184.27 ± 0.45 µg/g d.w.). This compound was also the most abundant among all detected bioactive compounds, consistent with previous studies [43,44]. Furthermore, caffeic acid was detected in both juices, with a higher concentration in SJ, corroborating prior reports that identified this phenolic acid as one of the dominant compounds in sage juice [14]. Similarly, phenolic acids were previously reported as the dominant class of bioactive compounds in peppermint extracts [21].

Chrysoeriol, a flavonoid known for its antimicrobial and anti-inflammatory properties, was detected in SJ [45]. Additionally, this compound has demonstrated antidiabetic potential *in vitro* and in animal models [45]. Another biologically active flavonoid glucoside, luteolin-7-*O*-β-glucoside, was quantified only in SJ. Beyond its antioxidant and anti-inflammatory activities, this compound has been shown to improve insulin resistance in mice with diet-induced obesity [46].

Hyperoside, another flavonoid detected in SJ, has been linked to various health benefits, including antioxidant, antiviral, antidiabetic, anti-inflammatory, antiproliferative, lipid-lowering, and cardioprotective properties [15,46,47,48]. Moreover, recent studies suggest that flavonoids like hyperoside and luteolin-7-*O*-β-glucoside can influence the composition and function of the intestinal microbiota, promoting beneficial bacterial growth and improving gut health, which may contribute to their systemic effects, including metabolic regulation [49].

Overall, the findings of this study suggest that SJ contains a higher concentration of phenolic compounds than PJ, leading to superior biological effects, including antioxidant, antimicrobial, anti-inflammatory, antidiabetic, and anti-allergic properties [4,47]. The potential interactions between these bioactive compounds and the gut microbiota warrant further investigation, as polyphenols are known to modulate microbial communities, which in turn can enhance their bioavailability and biological efficacy [50].

As noted by Rašeta et al. [51], variations in phenolic profiles can be attributed to multiple factors, such as the temporal stability of phenolics, environmental conditions, extraction methods, and the analytical techniques used for quantification. These findings highlight the potential application of SJ as a rich source of polyphenolic compounds—particularly phenolic acids, flavanols, and flavonoids—positioning it as a promising antioxidant and antidiabetic agent.

### 3.2. Antidiabetic Effects

The BW of normoglycemic animals during 10-day treatment with either saline (10 mL/kg BW) or SJ or PJ at doses of 20 mg/kg BW, 40 mg/kg BW, and 80 mg/kg BW, administered once daily, is shown in Table S3. The initial BW values of the tested groups were not statistically different.

During the observed period an increase in BW was detected in the control and all experimental groups, though the differences in BW increase were not significant.

The oral glucose tolerance test (OGTT) was conducted on healthy animals before treatment and on days 5 and 10 of treatment (Table 2).

Throughout the treatment period, no statistically significant differences in blood glucose levels (ΔBGL) were observed between animals treated with saline and those treated with SJ or PJ. After 10 days of treatment, a reduced increase in BGL was detected during OGTT in the experimental groups compared to the control group treated with saline, although the effect was not statistically significant. This suggests that a longer treatment duration may be required for the anti-hyperglycemic effect to fully manifest in healthy animals.

Table S4 presents the BW of animals with streptozotocin-induced diabetes during the 10-day treatment with either saline (10 mL/kg BW) or SJ or PJ at doses of 20 mg/kg BW, 40 mg/kg BW, or 80 mg/kg BW, administered once daily. The initial BW values among the tested groups were not statistically different. A slight increase in BW was observed in the control group and most experimental groups, except for the groups treated with the highest doses of SJ and PJ (SJ80b, PJ80b), in which a decrease in BW was noted.

The blood glucose levels of the mice before and after streptozotocin administration, followed by a 10-day treatment with either saline (10 mL/kg BW) or SJ or PJ at the tested doses, are shown in Table 3.

There were no statistically significant differences in glycemia values between the groups (C_2_, SJ20b–PJ80b) before streptozotocin administration. During the 10-day treatment period, BGL increased in all groups, with highest increase being observed in the saline-treated group (13.65 ± 7.74 mmol/L). In contrast, the increase in BGL in most groups treated with SJ or PJ was reduced twofold compared to the saline group, being lowest in the groups treated with the lowest doses of the juices (SJ20b, PJ20b). However, due to high variability in the measured values, the differences between the blood glucose levels were not statistically significant.

The antidiabetic effects of different sage and peppermint extracts have been verified in various models. Bayani et al. [52] reported that the administration of peppermint water extract in diabetic rats resulted in both hypoglycemic and hypocholesterolemic effects comparable to those of glibenclamide. Similarly, Ben Khedher et al. [17] demonstrated that sage extract exerted an antidiabetic effect comparable to rosiglitazone, with the proposed mechanism being increased insulin sensitivity. Other studies suggest that additional mechanisms may be involved. Lima et al. [14] found that sage tea administration in mice enhanced glucose uptake in the liver and reduced gluconeogenesis, exerting a metformin-like effect. Several reports indicate that both sage and peppermint are valuable sources of bioactive compounds with beneficial effects on diabetes, including glucose and lipid metabolism regulation [14,35,53,54]. The findings of our study align with these previous reports, suggesting that a simple juice preparation method can effectively extract these bioactive compounds [52,55]. Recently, Al-Mijalli et al. [26] confirmed that *M. piperita* essential oils can exhibit *in vitro* antidiabetic activity by inhibiting both α-amylase and α-glucosidase. Moreover, Oalđe Pavlović et al. [56] tested three types of extracts from both species, commercially cultivated in Serbia, and recorded significant *in vitro* α-glucosidase inhibitory activity. Additionally, they conducted an *in silico* analysis and found that among the quantified secondary metabolites, kaempferol-3-*O*-glucoside exhibited the highest binding energy for α-glucosidase inhibition, followed by isoquercetin, rutin, and naringin. Their results also showed that most secondary metabolites had lower binding energies than the positive control, acarbose, suggesting stronger and more efficient molecular binding to the enzyme. Recently, Sun et al. [57] summarized that polyphenols present in SJ and PJ may influence gut microbiota composition, potentially enhancing their antidiabetic effects.

### 3.3. Effect on Liver Function Biochemical Parameters

Liver damage is commonly indicated by the release of alanine aminotransferase (ALT) and aspartate aminotransferase (AST) into the serum. Since AST is also present in cardiomyocytes, neurons, nephrons, myocytes, and red blood cells, ALT serves as a more specific biomarker of liver damage [58].

Apart from the PJ40a group, AST and ALT levels were lower in all groups treated with SJ or PJ compared to the saline-treated group. The most pronounced reductions were observed in the groups receiving the lowest doses of SJ or PJ. However, due to high standard deviation (SD) values, none of these differences were statistically significant compared to the negative control group, despite the observed reductions in AST and ALT levels (Table 4). This finding aligns with the previously mentioned results, providing additional evidence for the protective effects of the tested extracts against oxidative damage.

To the best of our knowledge, there are no comparable data on hepatoprotection through treatment with SJ and PJ. However, similar findings in rat models have been reported by other research groups [55]. Additionally, Akdogan et al. [59] showed that the administration of *M. piperita* tea to Wistar albino rats led to a dose-dependent increase in ALT and AST activities, indicating potential liver damage. A recent study demonstrated significant *in vivo* hepatoprotective activity, showing reduced AST and ALT levels compared to the acetaminophen (AAP)-administered rat group, with a notable decrease in ALT (*p* < 0.05) observed for the four-week dried sage-based essential oil compared to the fresh herb-based oil; moreover, the enhanced hepatoprotective effects of the dried sage-based oil, both *in vivo* and *in vitro*, compared to other essential oil batches and the silymarin standard highlight the medicinal benefits of the drying protocol [36]. Treatment with hydroethanolic extracts of *S. officinalis* (250 mg/kg, intraperitoneally) for 28 days significantly reduced serum AST and ALT levels in rats co-administered with isoniazid (INH, 50 mg/kg) compared to the INH-only group. Biochemical and histopathological analyses showed a marked reduction in INH-induced hepatic damage at various plant concentrations. Hepatotoxicity was induced by administering INH (50 mg/kg, orally) for 28 days, with significant increases in serum transaminases indicating liver injury. Oxidative stress was implicated in the liver damage caused by INH [60].

### 3.4. Antioxidant Enzyme Activity

Superoxide dismutase (SOD) and catalase (CAT) play crucial roles as the first-line antioxidant defense mechanisms. The activity of these enzymes was enhanced in most of the groups treated with SJ or PJ. While antioxidant defense capacity decreased in animals treated with saline, significantly higher SOD activity was observed in the SJ20a, SJ80a, and PJ20a groups, while CAT activity was significantly elevated in the PJ20a group (*p* < 0.05) (Table 5).

The second line of the protective antioxidant defense system, consisting of glutathione peroxidase (GPx) and glutathione reductase (GR), was activated following depletion of the first-line defense enzymes. Higher GPx and GR activity levels were detected in the saline-treated group compared to most of the groups treated with SJ or PJ. Notably, a significantly lower GPx activity level was detected in the PJ40 group compared to the saline-treated group (*p* < 0.05) (Table 5).

Glutathione S-transferase (GST) activity was increased in most of the groups treated with SJ or PJ compared to the saline-treated group, with the increase being significant in the SJ20a and PJ20 groups compared to the saline group (*p* < 0.05) (Table 5). However, the effects of SJ and PJ on antioxidant enzyme activities did not follow a dose-dependent pattern.

The level of lipid peroxidation (LPx), a reliable marker for assessing the extent of cell membrane damage, was significantly lower in the majority of experimental groups treated with SJ or PJ compared to the saline-treated group (*p* < 0.05) (Table 5).

The observed antioxidant effects can likely be attributed to the rich content of dominant phenolic acids and flavonoid compounds present in the tested SJ and PJ. These bioactive compounds provide protection against reactive oxygen species by enhancing antioxidant capacity, which is considered a compensatory cellular response to oxidative damage [14]. Numerous studies have reported both *in vitro* [8,47] and *ex vivo* [59,61] antioxidant activity in different sage and peppermint extracts. In a study conducted by Akdogan et al. [59], it was shown that antioxidant enzyme activities, including SOD, GSH-Px, and CAT, were altered, reflecting changes in the oxidative stress response following the application of *M. piperita* tea.

### 3.5. Limitations of the Study

One of the potential limitations of this study is the fact that the traditionally prepared peppermint and sage juices contain sugar, which may affect their suitability for antidiabetic use. As shown in the results section, the lowest doses of both juices, which contain the least sugar, appeared to demonstrate the most effective anti-hyperglycemic effects. While our study suggests their antidiabetic potential, these findings indicate that modifying the traditional recipe could further improve their effects. Replacing sugar with alternative sweeteners, such as stevioside, could help maintain a satisfactory taste and may contribute to improved antidiabetic properties even when added in low doses [62], potentially making these juices more suitable for individuals with prediabetes or diabetes.

## 4. Conclusions

This study demonstrates that both sage juice (SJ) and peppermint juice (PJ) exert significant effects on glucose metabolism and antioxidant defense in experimental animals. Notably, the lowest dose (20 mg/kg BW) produced the most pronounced effects, with SJ and PJ at this concentration leading to the greatest reductions in blood glucose levels. PJ at the lowest dose also exhibited the highest antioxidant activity, while SJ displayed the most notable hepatoprotective effects at the same dose.

These findings suggest that traditionally prepared SJ and PJ are promising candidates for use as functional foods, with potential therapeutic applications in managing metabolic and oxidative stress-related disorders. Their appealing taste and bioactive properties make them suitable as dietary supplements. However, this is the first study to explore the biological effects of these juices, and further research is needed to confirm these results in human trials. Future studies should focus on optimizing dosage, examining long-term effects, and exploring the specific mechanisms through which these juices exert their beneficial effects.

## Figures and Tables

**Table 1:** Concentrations of selected phenolic compounds and quinic acid determined by LC-MS/MS analysis (µg/g dry weight).

Class	Compound	Sage Juice (SJ)	Peppermint Juice (PJ)
		µg/g d.w.	
Hydroxycinnamic acid	Caffeic acid	154.98 ± 0.01	16.96 ± 0.12
Cyclohexanecarboxylic acid	Quinic acid	2571.86 ± 1.15	184.27 ± 0.45
Flavonols	Luteolin-7-*O*-glucoside	110.72 ± 0.02	<LoQ
	Quercetin-3-*O*-galactoside	40.68 ± 0.25	<LoQ
Flavones	Apigenin	16.40 ± 1.01	<LoQ
	Apigenin-7-*O*-glucoside	324.36 ± 1.15	<LoQ
	Chrysoeriol	3.29 ± 0.13	<LoQ

Values are presented as the means ± SD of three measurements. <LOQ indicates a detected compound with a peak observed at a concentration lower than the limit of quantification (LOQ) but higher than the limit of detection (LOD). The following abbreviations are used for the examined parameters: d.w.—dry weight.

**Table 2:** Blood glucose levels (BGL) from OGTT (mmoL/L ± SEM).

	BGL 0 Before	BGL 0 After	Δ BGL 0	BGL After 5-Day Treatment Before	BGL After 5-Day Treatment After	Δ BGL 5	BGL After 10-Day Treatment Before	BGL After 10-Day Treatment After	Δ BGL 10
C_1_ (saline)	4.30 ± 0.42	7.55 ± 1.14	3.25 ± 0.83	6.60 ± 1.07	8.75 ± 1.39	2.15 ± 1.45	7.72 ± 0.74	9.55 ± 1.83	1.83 ± 1.45
SJ-20a	4.62 ± 0.68	7.57 ± 1.55	2.95 ± 1.38	6.18 ± 1.05	9.75 ± 2.57	3.57 ± 1.62	7.50 ± 1.60	8.15 ± 1.40	0.65 ± 0.68
SJ-40a	4.58 ± 0.51	8.58 ± 1.15	4.00 ± 1.23	6.67 ± 0.86	8.98 ± 1.35	2.32 ± 0.75	8.60 ± 1.12	9.97 ± 1.09	1.37 ± 0.38
SJ-80a	4.63 ± 0.92	8.40 ± 1.81	3.77 ± 1.52	6.55 ± 0.66	9.03 ± 1.03	2.48 ± 0.80	8.58 ± 0.91	9.88 ± 1.39	1.30 ± 0.64
PJ-20a	4.50 ± 0.80	7.73 ± 1.26	3.23 ± 0.68	6.25 ± 1.18	9.10 ± 2.03	2.85 ± 0.91	8.08 ± 1.42	9.40 ± 2.00	1.32 ± 1.25
PJ-40a	4.17 ± 0.79	7.63 ± 0.23	3.47 ± 0.80	6.50 ± 0.78	9.90 ± 1.36	3.40 ± 1.00	7.85 ± 0.78	8.83 ± 1.61	0.98 ± 0.99
PJ-80a	4.00 ± 0.33	6.53 ± 1.13	2.53 ± 1.11	6.02 ± 0.91	8.17 ± 1.49	2.15 ± 0.86	7.78 ± 1.86	9.17 ± 1.63	1.38 ± 1.32

Values are presented as the means ± SEM of six measurements. The following abbreviations are used for the examined parameters: BGL—blood glucose levels; PJ—peppermint juice; SJ—sage juice; SEM—standard error of the mean; C_1_—control group, which includes the administration of saline; 20a—treatment with 20 mg/kg BW (biological replicate a); 40a—treatment with 40 mg/kg BW (biological replicate a); 80a—treatment with 80 mg/kg BW (biological replicate a).

**Table 3:** Blood glucose levels (BGL) of animals with streptozotocin-induced diabetes (mmoL/L ± SD).

	BGL Start	BGL 72 h After STZ Administration	BGL After 5-Day Treatment	BGL After 10-Day Treatment	Δ BGL 10–72 h
C_2_ (saline)	6.92 ± 0.80	15.89 ± 3.45	22.42 ± 7.66	29.54 ± 8.94	13.65 ± 7.74
SJ-20b	6.31 ± 1.15	16.25 ± 7.33	20.89 ± 10.19	21.50 ± 10.38	5.25 ± 5.56
SJ-40b	6.19 ± 0.97	17.19 ± 7.24	25.15 ± 9.47	24.84 ± 7.88	7.65 ± 9.80
SJ-80b	5.54 ± 1.54	23.01 ± 7.46	28.10 ± 6.80	29.96 ± 5.28	6.95 ± 6.45
PJ-20b	5.48 ± 1.33	15.14 ± 4.80	21.46 ± 9.59	20.60 ± 7.75	5.46 ± 8.64
PJ-40b	5.95 ± 1.30	16.29 ± 5.13	20.19 ± 8.33	22.82 ± 7.28	6.54 ± 4.53
PJ-80b	5.65 ± 0.87	21.61 ± 7.68	28.02 ± 6.94	27.38 ± 6.80	5.76 ± 5.05

Values are presented as the means ± SD of six measurements. The following abbreviations are used for the examined parameters: BGL—blood glucose levels; PJ—peppermint juice; SJ—sage juice; STZ—streptozocin; SD—standard deviation; C_2_—control group, which includes the administration of saline; 20b—treatment with 20 mg/kg BW (biological replicate b); 40b—treatment with 40 mg/kg BW (biological replicate b); 80b—treatment with 80 mg/kg BW (biological replicate b).

**Table 4:** Alanine aminotransferase and aspartate aminotransferase levels (IU/L ± SD).

	AST	ALT
C_1_ (saline)	7025.8 ± 1038.8	10679 ± 1409.2
SJ-20a	2782 ± 426.9	5336.5 ± 801.5
SJ-40a	4784.3 ± 893.3	7707.2 ± 725.6
SJ-80a	5835.3 ± 1055.1	8481 ± 933.4
PJ-20a	5113.3 ± 932.7	8112.8 ± 1079.3
PJ-40a	8410 ± 1988.3	12,904 ± 2365.4
PJ-80a	6920.8 ± 1326.1	10,010 ± 1665.8

Values are presented as the means ± SD of six measurements. The following abbreviations are used for the examined parameters: PJ—peppermint juice; SJ—sage juice; SD—standard deviation; C_1_—control group, which includes the administration of saline; AST—asparatate aminotransferase; ALT—alanine aminotransferase; 20a—treatment with 20 mg/kg BW (biological replicate a); 40a—treatment with 40 mg/kg BW (biological replicate a); 80a—treatment with 80 mg/kg BW (biological replicate a).

**Table 5:** *Ex vivo* antioxidant enzyme activity after ten-day administration of saline/plant juices followed by paracetamol administration (130 mg/kg BW), presented as mean ± SD (n = 6).

	SOD U/mg of Proteins	CAT U/mg of Proteins	GPx nmol NADPH/min/mg Proteins	GR nmol NADPH/min/mg Proteins	GST nmol of Conjugate/min/mg or Proteins	LPx nmol MDA/mg of Proteins
C_1_ (saline)	18.83 ± 2.04	25.00 ± 2.04	37.54 ± 4.91	5.61 ± 0.83	18.73 ± 8.97	0.066 ± 0.019
SJ-20a	24.17 ± 2.86 *	27.39 ± 1.90	36.66 ± 3.86	4.90 ± 0.44	33.86 ± 5.41 *	0.048 ± 0.008 *
SJ-40a	19.78 ± 3.85	24.00 ± 4.73	32.94 ± 3.48	5.55 ± 0.99	23.37 ± 6.32	0.051 ± 0.005 *
SJ-80a	23.85 ± 5.54 *	26.27 ± 6.89	37.34 ± 5.81	5.47 ± 1.02	23.09 ± 8.62	0.054 ± 0.006
PJ-20a	30.15 ± 3.99 *	30.29 ± 2.09 *	38.39 ± 3.55	5.36 ± 0.65	29.30 ± 7.26 *	0.078 ± 0.01
PJ-40a	22.46 ± 2.52	25.35 ± 2.27	31.18 ± 2.59 *	4.96 ± 0.67	18.09 ± 2.11	0.043 ± 0.013 *
PJ-80a	19.07 ± 2.44	22.82 ± 3.67	34.37 ± 3.14	5.41 ± 1.32	20.09 ± 6.01	0.043 ± 0.011 *

Values are presented as the means ± SD of six measurements. * *p* < 0.05 compared to saline. The following abbreviations are used for the examined parameters: PJ—peppermint juice; SJ—sage juice; SD—standard deviation; C_1_—control group, which includes the administration of saline; SOD—superoxide dismutase; CAT—catalase; GPx—glutathione peroxidase; GR—glutathione reductase; GST—glutathione S-transferase; LPx—lipid peroxidation; 20a—treatment with 20 mg/kg BW (biological replicate a); 40a—treatment with 40 mg/kg BW (biological replicate a); 80a—treatment with 80 mg/kg BW (biological replicate a).

## Data Availability

The original contributions presented in the study are included in the article/Supplementary Materials, further inquiries can be directed to the corresponding author.

## Supplementary Materials

The following supporting information can be downloaded at: https://www.mdpi.com/article/10.3390/foods14071182/s1, Table S1: Optimized dynamic MRM parameters for seven quantified compounds^a^ in analyzed sage (SJ) and peppermint (PJ) juices; Table S2: Animal groups and treatment protocols; Table S3: Body weight of normoglycemic animals (g); mean ± SD, n = 6; Table S4: Body weight of animals with streptozotocin-induced diabetes (g); mean ± SD, n = 6.

## Abbreviations

The following abbreviations are used in this manuscript:

SJ	Sage juice
PJ	Peppermint juice
BW	Body weight
STZ	Streptozocin
LOD	Limit of detection
LOQ	Limit of quantification
LC-MS/MS	Liquid chromatography–mass spectrometry
OGTT	Oral glucose tolerance test
