# Supplementary material for: Health Benefits of Traditional Sage and Peppermint Juices: Simple Solutions for Antioxidant and Antidiabetic Support
Source: Foods. 2025 Mar 28;14(7):1182. doi: 10.3390/foods14071182 (PMC11988581; doi:10.3390/foods14071182)
Supplement: Supplementary file 1 [file foods-14-01182-s001.zip › foods-3519214-supplementary.pdf]

Table S1. Optimized dynamic MRM parameters for seven quantified compounds[a] in the analyzed sage (SJ) and peppermint (PJ) juices.

Class	Standard compounds	t_R [min]	Precursor *m/z*	Product *m/z*	$V_{fragmentor}$ (V)	$V_{collision}$ (V)
Hydroxycinnamic acids	Caffeic acid	1.19	179	135	100	10
Cyclohexanecarboxylic acid	Quinic acid	0.52	191	85	150	20
Flavonols	Luteolin-7-*O*-glucoside[a]	2.23	447	285	230	30
	Quercetin-3-*O*-galactoside[a]	2.16	463	300	200	30
Flavones	Apigenin[a]	4.71	269	117	130	25
	Apigenin 7-*O*-glucoside[a]	2.78	431	268	135	41
	Chrysoeriol[a]	4.82	299	284	125	20

[a] Values for the compounds shown in **Table 1** indicate a detected compound with a peak observed at a concentration lower than the limit of quantification (LOQ) but higher than the limit of detection (LOD).

Table S2. Animal groups and treatment protocols.

Group	Treatment (10 days/once per day)	Test
C_1(control group1)	saline 10 ml/kg BW	
SJ20a	sage juice 20 mg/kg BW	
SJ40a	sage juice 40 mg/kg BW	OGTT was performed on 5th and 10th day of the treatment
SJ80a	sage juice 80 mg/kg BW	
PJ20a	peppermint juice 20 mg/kg BW	
PJ40a	peppermint juice 40 mg/kg BW	
PJ80a	peppermint juice 80 mg/kg BW	
C_2(control group 2)	STZ 150 mg/kg + saline 10 ml/kg BW	
SJ20b	STZ 150 mg/kg + sage juice 20 mg/kg BW	
SJ40b	STZ 150 mg/kg + sage juice 40 mg/kg BW	Diabetes induced by STZ, and blood glucose level was measured on 5th and 10th day of the treatment
SJ80b	STZ 150 mg/kg + sage juice 80 mg/kg BW	
PJ20b	STZ 150 mg/kg + peppermint juice 20 mg/kg BW	
PJ40b	STZ 150 mg/kg + peppermint juice 40 mg/kg BW	
PJ80b	STZ 150 mg/kg + peppermint juice 80 mg/kg BW	

The following abbreviations are used for the examined parameters: SJ – Sage juice; PJ – peppermint juice; BW – body weight; OGTT – Oral glucose tolerant test; STZ – streptozotocin. 20a-b – Treatment with 20 mg/kg BW (biological replicates a and b); 40a-b – Treatment with 40 mg/kg BW (biological replicates a and b) 80a-b – Treatment with 80 mg/kg BW (biological replicates a and b).

Table S3. Body weight of normoglycemic animals (g).

	BW start	BW after 5 day treatment	BW after 10 day treatment	ΔBW
C_1 (saline)	36.83 ± 2.40	42.83 ± 3.54	41.00 ± 4.20	4.17 ± 2.71
SJ-20a	34.50 ± 2.43	40.50 ± 1.52	39.50 ± 1.38	5.00 ± 1.41
SJ-40a	32.66 ± 1.63	39.83 ± 1.72	38.30 ± 1.64	5.64 ± 0.75
SJ-80a	30.67 ± 2.16	36.50 ± 2.66	36.00 ± 2.37	5.33 ± 1.37
PJ-20a	32.17 ± 3.95	38.00 ± 4.24	38.17 ± 4.62	6.00 ± 2.19
PJ-40a	34.50 ± 2.35	40.67 ± 2.58	40.33 ± 2.50	5.83 ± 1.17

PJ-80a	37.83 ± 2.14	42.83 ± 2.64	41.17 ± 2.32	3.33 ± 1.51

Values are presented as means ± SD of three measurements. The following abbreviations are used for the examined parameters: SJ – Sage juice; PJ – peppermint juice; BW – body weight. 20a – Treatment with 20 mg/kg BW (biological replicate a); 40a – Treatment with 40 mg/kg BW (biological replicate a) 80a – Treatment with 80 mg/kg BW (biological replicate a).

Table S4. Body weight of animals with streptozotocin-induced diabetes (g).

	BW start	BW 72h after STZ administration	BW after 5 day treatment	BW after 10 day treatment	ΔBW 10-72h
C_2 (saline)	36.62±1.77	40.88±0.99	40.25±0.80	42.38±3.42	1.50±2.80
SJ-20b	36.88±1.89	39.75±2.12	40.25±2.19	41.00±2.04	1.25±2.79
SJ-40b	31.62±1.85	33.50±2.67	31.94±3.37	34.65±5.09	1.15±3.20
SJ-80b	33.12±2.90	37.12±2.80	36.62±2.43	36.88±3.18	-0.25±2.94
PJ-20b	30.62±1.19	32.25±1.67	32.25±1.77	33.75±2.58	1.50±2.80
PJ-40b	34.38±2.97	38.50±3.46	35.75±4.20	39.85±5.76	1.35±2.63
PJ-80b	33.50±1.77	33.25±2.43	33.00±2.79	32.94±3.46	-0.31±1.93

Values are presented as means ± SD of three measurements. The following abbreviations are used for the examined parameters: SJ – Sage juice; PJ – peppermint juice; BW – body weight; STZ – streptozotocin. 20b – Treatment with 20 mg/kg BW (biological replicate b); 40b – Treatment with 40 mg/kg BW (biological replicate b) 80b – Treatment with 80 mg/kg BW (biological replicate b).
